# Phosphatidic acid inhibits inositol synthesis by inducing nuclear translocation of kinase IP6K1 and repression of *myo*-inositol-3-P synthase

**DOI:** 10.1016/j.jbc.2022.102363

**Published:** 2022-08-10

**Authors:** Pablo Lazcano, Michael W. Schmidtke, Chisom J. Onu, Miriam L. Greenberg

**Affiliations:** Department of Biological Sciences, Wayne State University, Detroit, Michigan, USA

**Keywords:** phosphatidic acid, IP6K1, MIPS, valproate, lithium, inositol, AMPK, phospholipase D, inositol phosphate, glucose, AICAR, 5-aminoimidazole-4-carboxamide, AMPK, 5' AMP-activated protein kinase, ER, endoplasmic reticulum, FIPI, 5-fluoro-2-indolyl des-chlorohalopemide, IP6K1, inositol hexakisphosphate kinase, MEFs, mouse embryonic fibroblasts, MIPS, myo-inositol-3-phosphate synthase, NLS, nuclear localization signal, PA, phosphatidic acid, PLD, phospholipase D, PMA, phorbol 12-myristate 13-acetate, VPA, valproate

## Abstract

Inositol is an essential metabolite that serves as a precursor for structural and signaling molecules. Although perturbation of inositol homeostasis has been implicated in numerous human disorders, surprisingly little is known about how inositol levels are regulated in mammalian cells. A recent study in mouse embryonic fibroblasts demonstrated that nuclear translocation of inositol hexakisphosphate kinase 1 (IP6K1) mediates repression of *myo*-inositol-3-P synthase (MIPS), the rate-limiting inositol biosynthetic enzyme. Binding of IP6K1 to phosphatidic acid (PA) is required for this repression. Here, we elucidate the role of PA in IP6K1 repression. Our results indicate that increasing PA levels through pharmacological stimulation of phospholipase D (PLD) or direct supplementation of 18:1 PA induces nuclear translocation of IP6K1 and represses expression of the MIPS protein. We found that this effect was specific to PA synthesized in the plasma membrane, as endoplasmic reticulum–derived PA did not induce IP6K1 translocation. Furthermore, we determined that PLD-mediated PA synthesis can be stimulated by the master metabolic regulator 5′ AMP-activated protein kinase (AMPK). We show that activation of AMPK by glucose deprivation or by treatment with the mood-stabilizing drugs valproate or lithium recapitulated IP6K1 nuclear translocation and decreased MIPS expression. This study demonstrates for the first time that modulation of PA levels through the AMPK-PLD pathway regulates IP6K1-mediated repression of MIPS.

Inositol is a six-carbon cyclitol that is essential for all eukaryotic life (1). Deprivation of inositol results in “inositol-less death”, a phenotype originally described in yeast and supported by early work in human cell lines (2, 3, 4, 5, 6). The importance of inositol is due in part to its role as a precursor for other vital molecules, such as phosphoinositides, inositol polyphosphates, and pyrophosphates (1). Cells must obtain inositol through extracellular uptake *via* inositol transporters or *de novo* synthesis from glucose-6-phosphate.

Dysregulation of inositol metabolism has been linked to several prevalent human disorders, including Alzheimer’s, polycystic ovary syndrome, Lowe syndrome, various cancers, and bipolar disorder (7, 8, 9, 10). Furthermore, inositol depletion has been hypothesized as the mechanism of action of the mood-stabilizing drugs lithium and valproate (VPA), as both are known to inhibit the inositol biosynthetic pathway and thereby decrease inositol levels (11, 12, 13, 14). Despite its critical importance, little is known about the mechanisms that regulate inositol biosynthesis in mammalian cells.

Studies within the last decade have provided some of the first insights regarding the regulation of inositol synthesis in mammalian cells. Tissue-specific methylation was observed in the rat ISYNA1 gene, which encodes the rate-limiting enzyme in the inositol biosynthetic pathway, *myo*-inositol-3-phosphate synthase (MIPS) (15). This suggested that MIPS expression may be regulated by methylation. It was subsequently shown that inositol hexakisphosphate kinase 1 (IP6K1) modifies histone methylation patterns (16) and that upon binding with phosphatidic acid (PA), IP6K1 negatively regulates expression of MIPS by increasing methylation in the ISYNA1 promoter (17). Thus, these studies identified a potential link between ISYNA1 regulation, IP6K1, and PA.

PA can be generated through three distinct cellular pathways. These include (1) acylation of lyso-PA by lyso-PA acyltransferase, (2) phosphorylation of diacylglycerol by diacylglycerol kinase, and (3) hydrolysis of phosphatidylcholine by phospholipase D (PLD) (18, 19, 20, 21). PLD activity is regulated through phosphorylation by the highly conserved kinase 5′ AMP-activated protein kinase (AMPK) (22). As a master metabolic regulator, canonical activation of AMPK is mediated by an increase in the AMP/ADP:ATP ratio (23). Interestingly, the inositol-depleting drugs VPA and lithium have been shown to activate AMPK in multiple cell types, and VPA was shown to activate the AMPK homolog Snf1 in yeast (24, 25, 26, 27). Furthermore, a recent study identified inositol as a direct allosteric inhibitor of AMPK that acts by blocking activation by AMP (28). Taken together, these reports suggest a potential feedback mechanism involving PA, AMPK, IP6K1, and inositol synthesis.

Based on the above findings, we hypothesized that modulation of PA levels through the AMPK-PLD pathway serves as the regulatory mechanism underlying repression of inositol biosynthesis by IP6K1. In the current study, increasing PA levels by drug-mediated stimulation of PLD or activation of AMPK resulted in increased nuclear translocation of IP6K1 and decreased expression of MIPS. These findings delineate the first mechanistic model of regulation of inositol biosynthesis in mammalian cells in which PA controls IP6K1-mediated repression of MIPS by increasing IP6K1 localization in the nucleus.

## Results

### PA regulates IP6K1 localization

**Figure 1:**
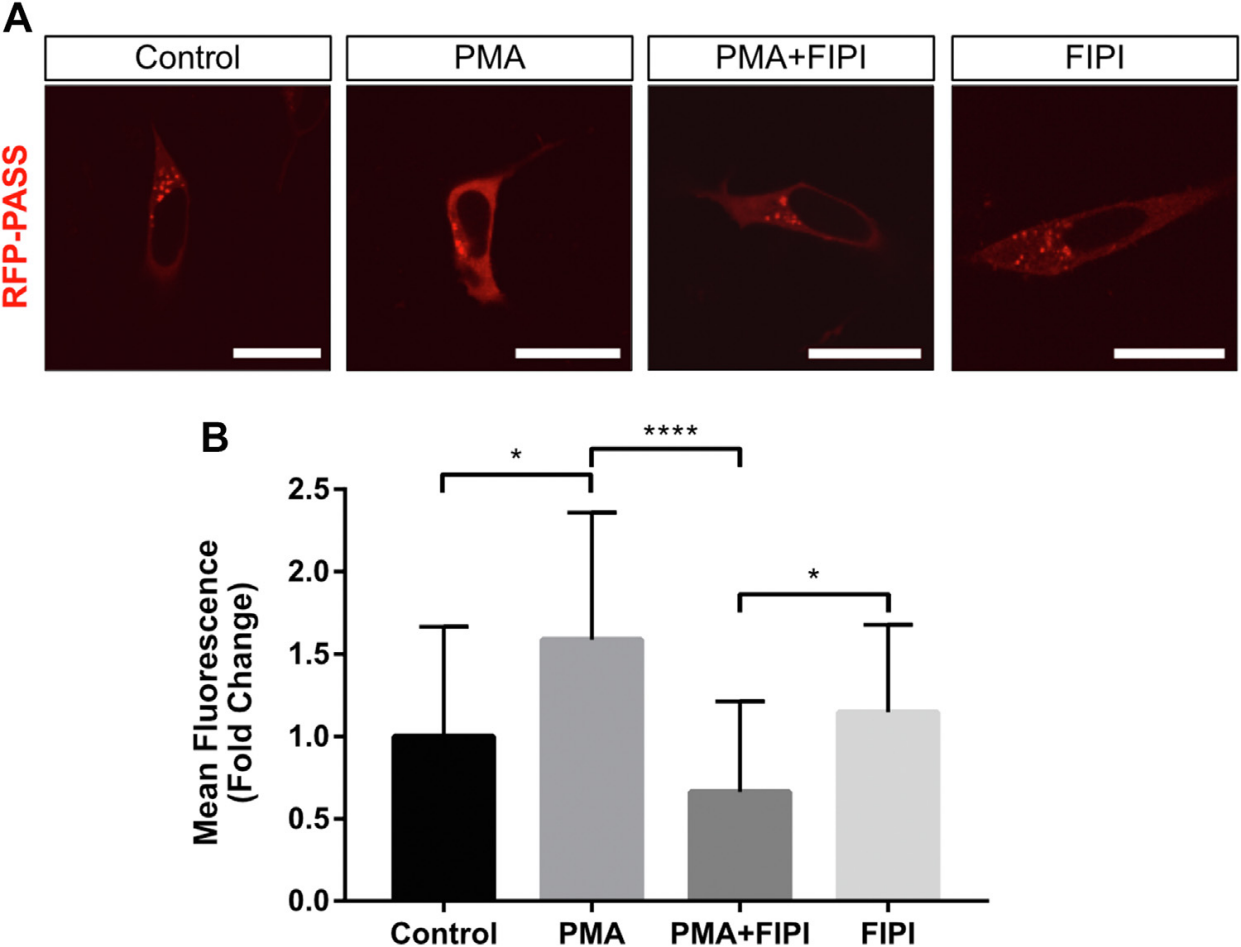
**PA levels are modulated by treatment with PMA or FIPI.***A*, cells transfected with RFP-PASS (*red fluorescence*) were treated with PMA alone (100 nM for 5 h) or in the presence of FIPI (0.75 μM, applied 30 min before PMA). n = 3 independent experiments, images are representative of the observed effect in most cells. The scale bars represent 20 μm. *B*, mean fluorescence quantification of the experiment depicted in (*A*). Arbitrary fluorescence units were normalized to untreated (*control*) cells and expressed as fold change ± SD. Statistical significance was analyzed by one-way ANOVA with a Tukey *post hoc* test, ∗*p* < 0.05, ∗∗∗∗*p* < 0.0001 where n = 3 independent experiments in which 20 to 30 cells were analyzed per condition. FIPI, 5-fluoro-2-indolyl des-chlorohalopemide; PA, phosphatidic acid; PMA, phorbol 12-myristate 13-acetate.

A previous study demonstrated that nuclear translocation of IP6K1 requires PA binding and that mRNA levels of the inositol biosynthetic enzyme MIPS are increased in IP6K1-KO mouse embryonic fibroblast (MEF) cells (17). To delineate the role of PA, we tested the hypothesis that modifying PA levels regulates MIPS expression by controlling IP6K1 localization. To modify PA levels, we targeted the PLD-mediated PA synthesis pathway, as PLD activity is amenable to pharmacological treatment and is endogenously regulated by AMPK, a putative target of inositol-depleting mood stabilizers (22, 28). MEF cells were treated with either phorbol 12-myristate 13-acetate (PMA), a drug known to induce PA synthesis through PKC-dependent activation of PLD (29, 30), or 5-fluoro-2-indolyl des-chlorohalopemide (FIPI), a potent PLD1/2 inhibitor (31). To confirm the efficacy of these treatments for modifying PA levels, the genetically encoded PA biosensors RFP-PASS and GFP-PASS were used to visually monitor changes in PA abundance (32). These constructs contain a strong PA-binding domain linked to either RFP or GFP as a fluorescent marker. MEF cells transfected with RFP-PASS were treated with 100 nM PMA for 5 h in the presence or absence of 0.75 μM FIPI and analyzed by confocal microscopy. As expected, PMA treatment induced PA synthesis, as evidenced by an approximately 1.5-fold increase in RFP fluorescence at the plasma membrane (Fig. 1). Pretreatment with FIPI blocked the increase in RFP fluorescence, confirming that the effect of PMA on PA levels is PLD dependent (Fig. 1).

**Figure 2:**
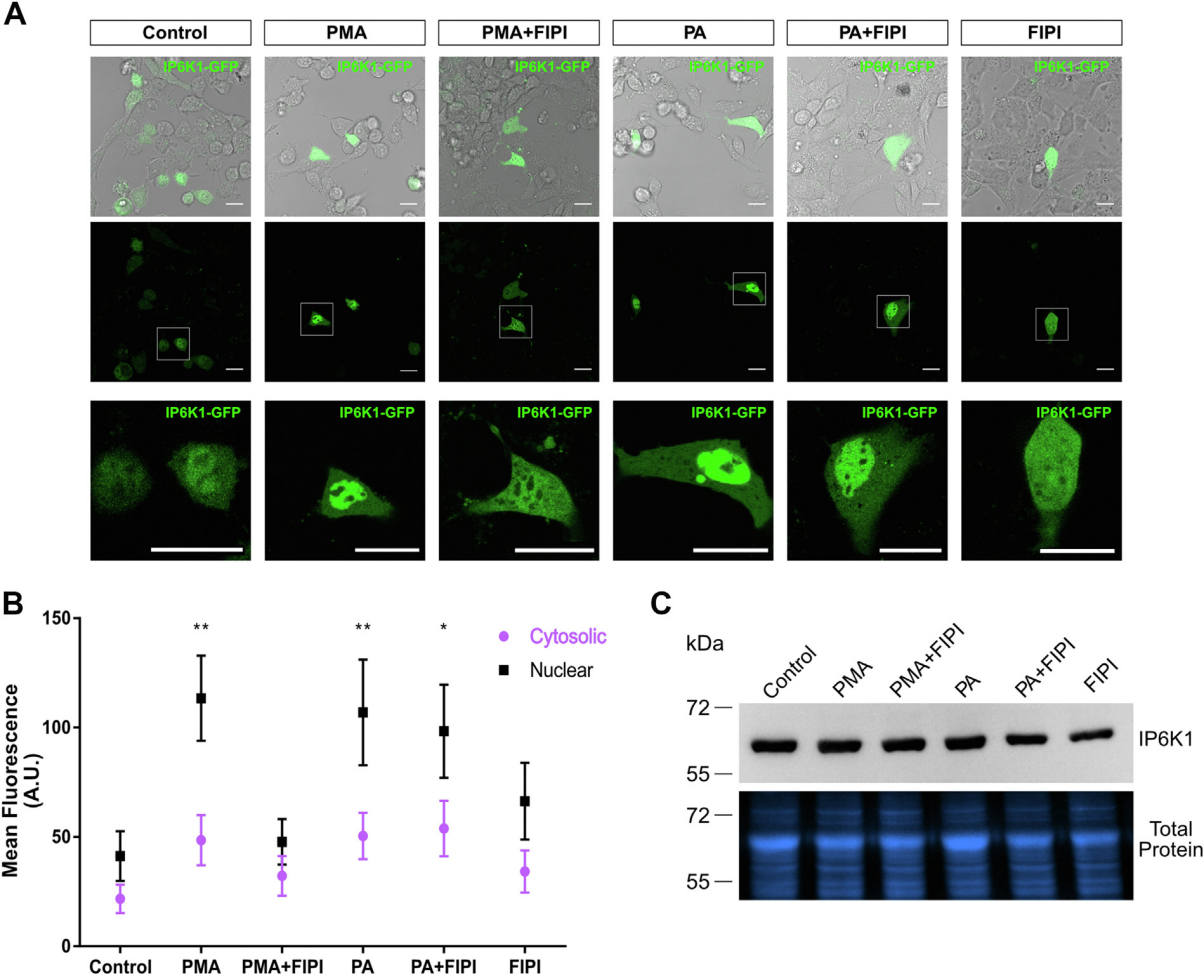
**IP6K1 localization is regulated by PA.***A*, IP6K1-KO cells transfected with IP6K1-GFP (*green*) were treated with 100 nM PMA for 16 h or 100 μM 18:1 PA for 5 h, in the presence or absence of FIPI (0.75 μM, applied 30 min before PMA or PA). *Top* panel: merged image of transmitted light with *green fluorescence* image to evaluate transfection efficiency. *Middle* panel: *green fluorescence* alone; the boxed region is magnified to show further detail in the *lower* panel. The scale bars represent 20 μm. *B*, mean fluorescence (arbitrary units, A.U.) of cytosolic and nuclear sections of cells as shown in (*A*). Statistical significance was analyzed by a Kolmogorov–Smirnov test, ∗*p* < 0.05, ∗∗*p* < 0.01 where n = 3 independent experiments in which 20 to 30 cells were analyzed per condition. *C*, representative Western blot analysis of IP6K1 in WT MEF cells treated as in (*A*). n = 3 independent experiments, image is representative of the effect observed in all the experiments. Total protein staining was used to normalize for protein loading. FIPI, 5-fluoro-2-indolyl des-chlorohalopemide; IP6K1, inositol hexakisphosphate kinase; MEF, mouse embryonic fibroblast; PA, phosphatidic acid; PMA, phorbol 12-myristate 13-acetate.

To track IP6K1 localization, IP6K1-KO MEF cells were transfected with an IP6K1-GFP expression plasmid (17). This approach ensured that all IP6K1 in the cell could be visualized by GFP fluorescence. To modify PA levels, cells were treated with PMA or cell-permeable 18:1 PA (33) in the presence or absence of FIPI, then localization of IP6K1 was monitored by confocal microscopy. IP6K1 is normally localized in both the cytosol and nucleus; however, following treatment with either PMA or 18:1 PA, nuclear localization of IP6K1 was notably increased (Fig. 2, *A* and *B*). As expected, pretreatment with FIPI prevented the change in IP6K1 localization in PMA-treated cells but not in cells supplemented with exogenous 18:1 PA, which accumulate PA independent of PLD activity (Fig. 2, *A* and *B*). Total IP6K1 protein levels were unaffected by either treatment (Fig. 2*C*). These results demonstrate that MEF cells accumulate IP6K1 in the nucleus as a function of PA levels.

PA is synthesized in multiple subcellular locations, and the acyl chain composition of newly synthesized PA is influenced by the site of synthesis, reflecting differences in the composition of available precursor lipids from which PA is synthesized (34, 35, 36). The source of PA is critical for its regulatory functions. For example, it was recently demonstrated that only PA synthesized in the plasma membrane (but not the endoplasmic reticulum (ER), Golgi, or endosomes) can regulate Hippo pathway signaling (37, 38). Therefore, to test whether the source of PA affects its ability to induce IP6K1 translocation, an optogenetic approach (optoPLD) was adopted wherein PA synthesis by PLD can be targeted to specific subcellular membranes and temporally regulated by exposure to blue light (38).

**Figure 3:**
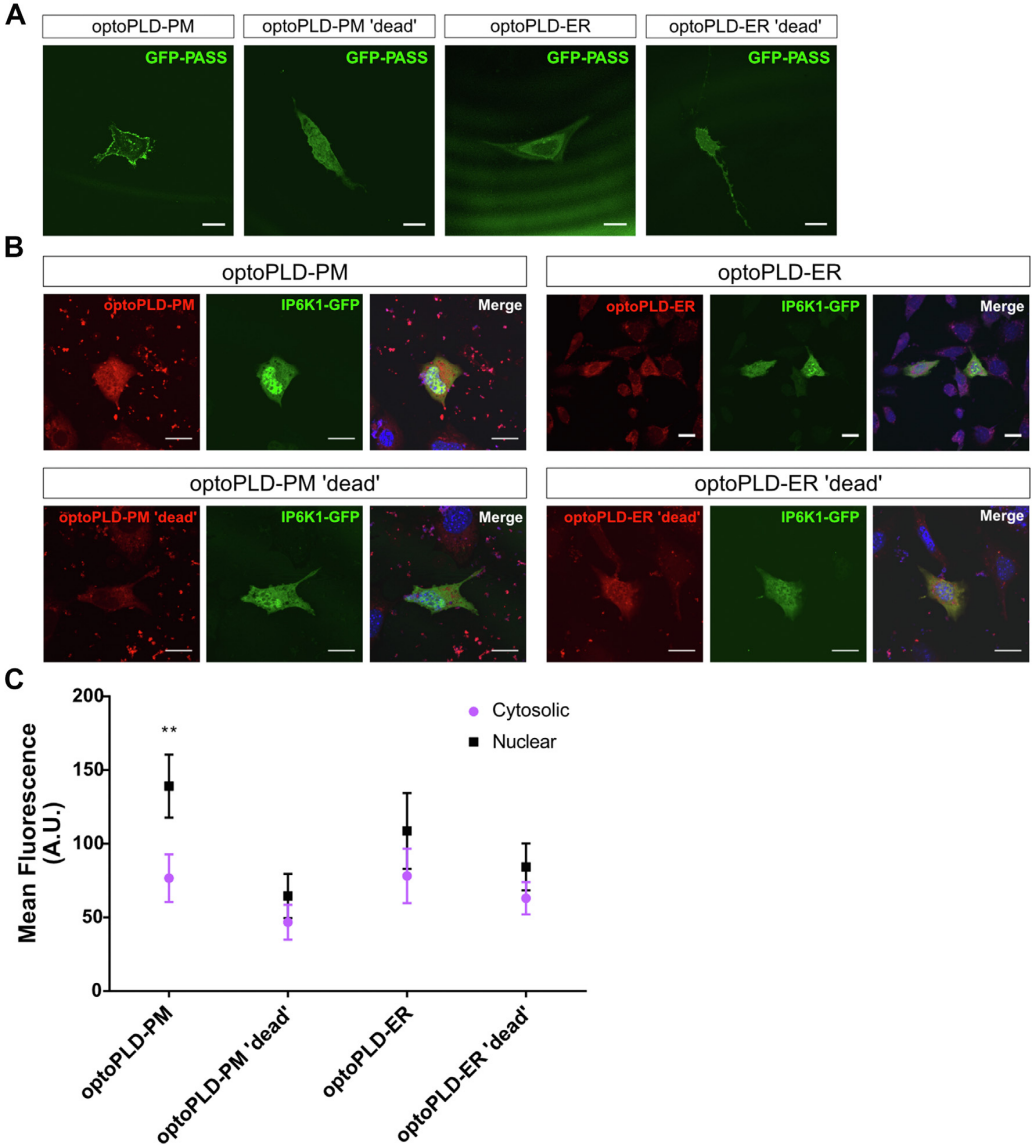
**Plasma membrane–derived PA induces IP6K1 nuclear localization.***A*, MEF cells were cotransfected with GFP-PASS (*green*) and either optoPLD-PM, optoPLD-PM ‘dead’, optoPLD-ER, or optoPLD-ER ‘dead’ as indicated. Cells were stimulated using intermittent blue light with a 5-s pulse every 2 min for a total of 30 min. PA localization was evaluated by GFP-PASS fluorescence using confocal microscopy. The scale bar represents 20 μm. Images are representative of the observed phenotype. *B*, IP6K1-KO MEF cells were cotransfected with IP6K1-GFP (*green*) and optoPLD-PM (*red, top left*), optoPLD-ER (*red, top right*), optoPLD-PM ‘dead’ (*red, bottom left*), or optoPLD-ER ‘dead’ (*red, bottom right*). Cells were stimulated using intermittent blue light with a 5-s pulse every 2 min for a total of 30 min. IP6K1 localization was determined by confocal microscopy. DAPI was used as nuclear staining. The scale bar represents 20 μm. *C*, mean fluorescence (arbitrary units, A.U.) of cytosolic and nuclear sections of cells as shown in (B). Statistical significance was analyzed by a Kolmogorov–Smirnov test, ∗∗*p* < 0.01 where n = 3 independent experiments in which 20 to 30 cells were analyzed per condition. IP6K1, inositol hexakisphosphate kinase; MEF, mouse embryonic fibroblast; ER, endoplasmic reticulum; PA, phosphatidic acid; PLD, phospholipase D; PMA, phorbol 12-myristate 13-acetate.

Using this approach, PA synthesized at the plasma membrane (PM-optoPLD) was compared with ER-derived PA (ER-optoPLD) for its ability to stimulate nuclear localization of IP6K1. Cotransfection of the PA biosensor GFP-PASS with either PM-optoPLD or ER-optoPLD confirmed proper targeting of PA synthesis to the PM and perinuclear ER, respectively (Fig. 3*A*). This effect did not occur using the corresponding catalytically ‘dead’ control constructs. Interestingly, only PM-derived PA (but not ER-derived PA) was able to stimulate IP6K1 nuclear translocation (Fig. 3, *B* and *C*). As expected, nuclear localization was not stimulated by the catalytically ‘dead’ constructs. Collectively, these findings further support the hypothesis that PA regulates nuclear translocation of IP6K1 and indicate that PA synthesized by PLD in the PM is unique in its ability to mediate this effect.

### PA regulates MIPS expression

**Figure 4:**
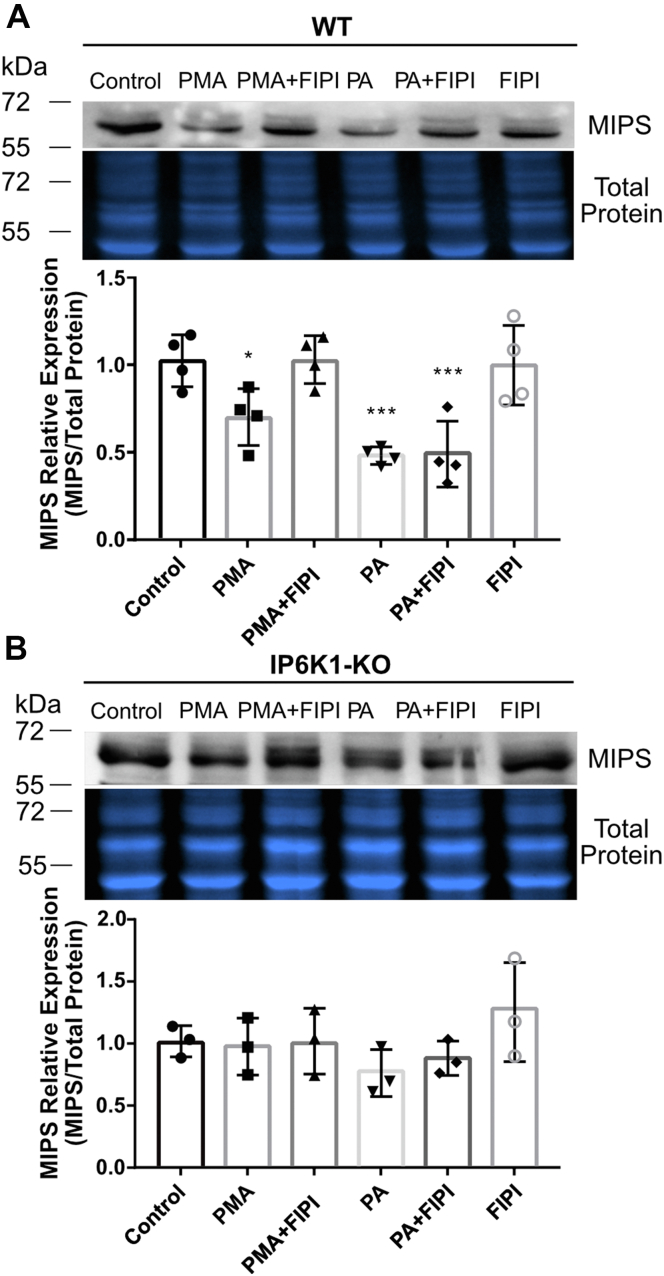
**MIPS expression is regulated by PA in the presence of IP6K1.***A*, Western blot against MIPS protein in WT MEF cells treated with 100 nM PMA for 16 h or 100 μM 18:1 PA for 5 h, in the presence or absence of FIPI (0.75 μM, applied 30 min before PMA or PA). *B*, Western blot against MIPS protein in IP6K1-KO MEF cells treated with 100 nM PMA for 16 h or 100 μM 18:1 PA for 5 h, in the presence or absence of FIPI (0.75 μM, applied 30 min before PMA or PA). Graphs depict band quantification. Total protein staining was used to normalize for protein loading. n = 4 independent experiments for (*A*) and n = 3 independent experiments for (*B*). Statistical significance was analyzed by one-way ANOVA with a Tukey *post hoc* test, ∗*p* < 0.05 and ∗∗∗*p* < 0.001. FIPI, 5-fluoro-2-indolyl des-chlorohalopemide; IP6K1, inositol hexakisphosphate kinase; MEF, mouse embryonic fibroblast; MIPS, *myo*-inositol-3-phosphate synthase; PA, phosphatidic acid; PMA, phorbol 12-myristate 13-acetate.

Yu *et al.* showed that IP6K1-KO cells have higher levels of MIPS mRNA and protein than WT MEF cells, suggesting that IP6K1 serves as a negative regulator of MIPS expression (17). To test the hypothesis that PA levels modulate MIPS expression by controlling IP6K1 localization, we treated cells with PMA in the presence or absence of FIPI and measured MIPS protein levels by Western blot. Treatment with either PMA or 18:1 PA resulted in a significant reduction in MIPS protein (Fig. 4*A*), and as expected, only the PMA-induced decrease was blocked by pretreatment with FIPI (Fig. 4*A*). Strikingly, regulation of MIPS protein levels by PA did not occur in IP6K1-KO MEF cells, indicating that IP6K1 is required for this regulatory mechanism to function (Fig. 4*B*). Taken together, these results show that MIPS expression is regulated by PA levels in an IP6K1-dependent manner.

### Increasing PA levels by glucose deprivation induces nuclear translocation of IP6K1 and MIPS repression

**Figure 5:**
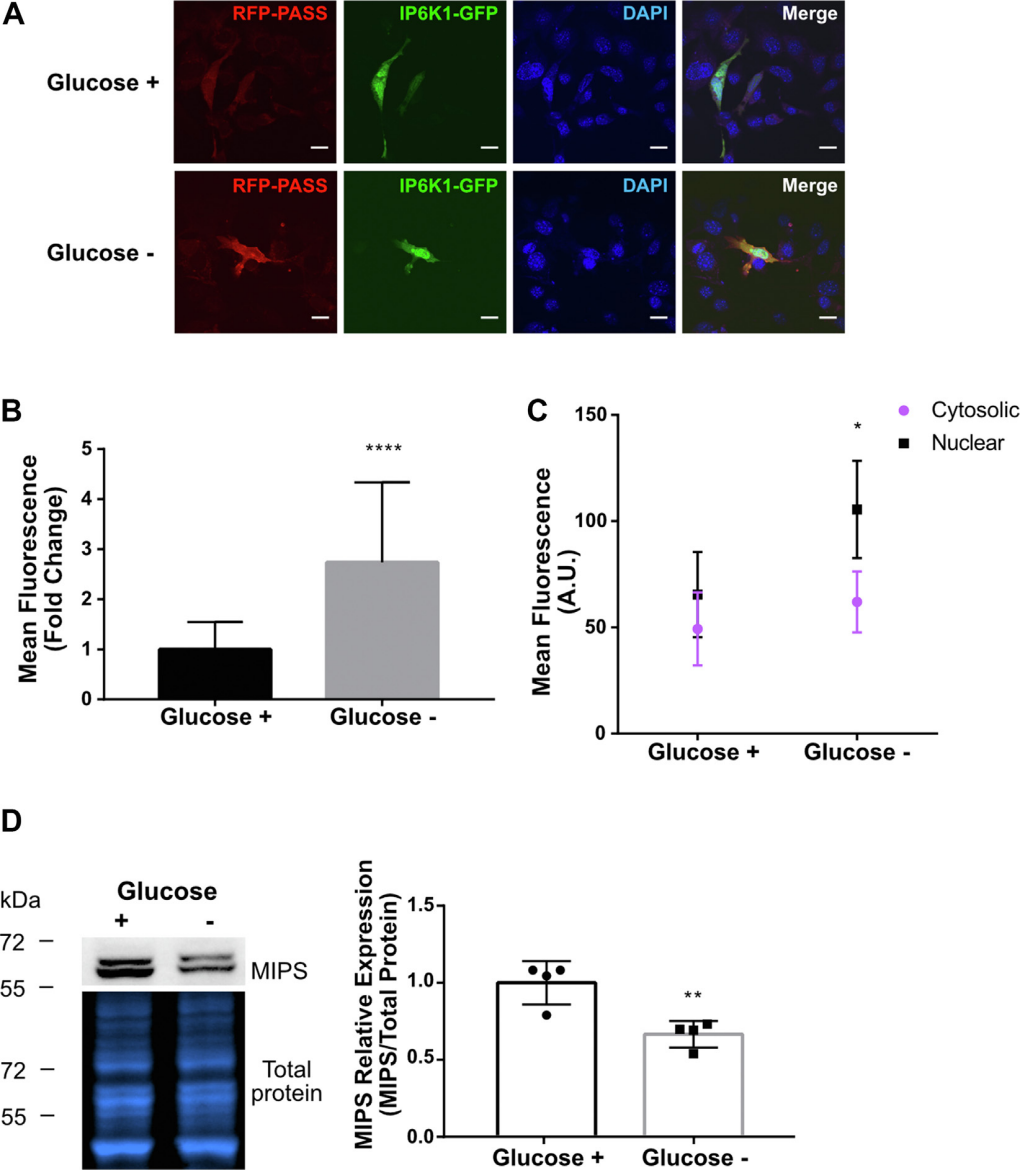
**Glucose deprivation induces PA synthesis, IP6K1 nuclear translocation, and MIPS repression.***A*, IP6K1-KO MEF cells were cotransfected with IP6K1-GFP and RFP-PASS. Cells were deprived of glucose for 16 h, and PA synthesis and IP6K1 localization were monitored by confocal microscopy. DAPI was used to stain nuclei. The scale bar represents 20 μm. *B*, mean fluorescence quantification of RFP-PASS from the experiment depicted in (*A*). Arbitrary fluorescence units (A.U.) were normalized to control and expressed as fold change ± SD. Statistical significance was analyzed by an unpaired *t* test, ∗∗∗∗*p* < 0.0001, where n = 3 independent experiments and between 20 to 30 cells were analyzed per condition. *C*, mean fluorescence (A.U.) of peripheral cytosolic and nuclear sections of cells as shown in (*A*). Statistical significance was analyzed by a Kolmogorov–Smirnov test, ∗*p* < 0.05 where n = 3 independent experiments and between 20 to 30 cells were analyzed per condition. *D*, Western blot against MIPS protein in WT MEF cells grown in control conditions (glucose +) or deprived of glucose for 16 h (glucose −). Graphs depict band quantification (*right*). Total protein staining was used to normalize for protein loading. n = 4 independent experiments for each condition. Statistical significance was analyzed by an unpaired *t* test, ∗∗*p* < 0.01. IP6K1, inositol hexakisphosphate kinase; MEF, mouse embryonic fibroblast; MIPS, *myo*-inositol-3-phosphate synthase; PA, phosphatidic acid.

The above findings utilized drug treatments and exogenous supplementation of PA to alter intracellular PA levels. To complement these experiments, we took an orthogonal approach to assay nuclear translocation of IP6K1 under physiological conditions that stimulate PA synthesis. PLD1-mediated PA synthesis is increased in response to activation of the highly conserved kinase AMPK (22). AMPK is widely recognized as a master metabolic regulator that is activated by phosphorylation when energy is low to shift cells from anabolism to catabolism (23, 39). Numerous studies reported increased AMPK activation in response to glucose limitation (40, 41, 42, 43). Therefore, to determine the effect of energy stress-induced PA synthesis on the localization of IP6K1, cells were grown in glucose-free conditions for 16 h and PA abundance and IP6K1 localization were monitored. As seen in Figure 5, *A* and *B*, glucose-deprived cells exhibited a 3-fold increase in RFP-PASS fluorescence, indicative of increased PA synthesis. Increased PA correlated with an increase in the nuclear localization of IP6K1 (Fig. 5*C*) and a significant reduction in MIPS protein levels (Fig. 5*D*).

### Activation of AMPK by lithium or VPA induces nuclear localization of IP6K1 and MIPS repression

**Figure 6:**
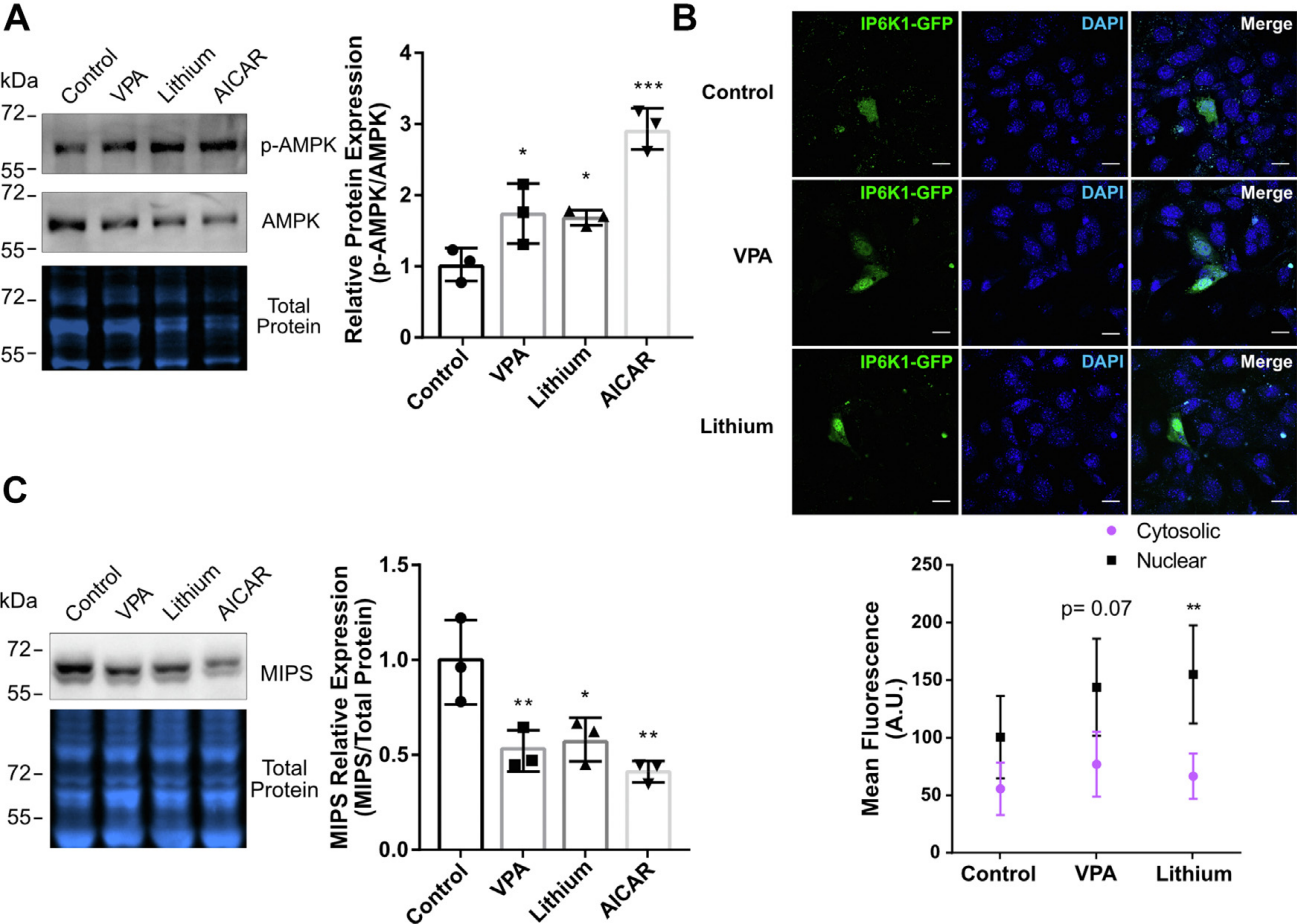
**VPA and lithium activate AMPK, induce IP6K1 nuclear translocation, and repress MIPS protein expression.***A*, cells were treated with 1 mM VPA or 10 mM lithium for 24 h or 500 μM AICAR for 1 h, and AMPK activation was determined by Western blot. Samples were probed for p-AMPK, stripped until no signal was detected, and reprobed for total AMPK. Total protein staining was used to normalize for protein loading. The graph shows the relative protein expression of p-AMPK normalized against total AMPK and then total protein. Statistical significance was analyzed by parametric *t* test, ∗*p* < 0.05 and ∗∗∗*p* < 0.001, where n = 3 independent experiments. *B*, cells were treated with either 1 mM VPA or 10 mM lithium for 24 h and IP6K1 localization was determined by fluorescence confocal microscopy. DAPI was used as nuclear staining. The scale bar represents 20 μm. Graph (*lower panel*) shows mean fluorescence (arbitrary units, A.U.) of cytosolic and nuclear sections of cells. Statistical significance was analyzed by a Kolmogorov–Smirnov test, ∗∗*p* < 0.01 where n = 3 independent experiments in which 20 to 30 cells were analyzed per condition. *C*, Western blot against MIPS protein in WT MEF cells treated with either 1 mM VPA, 10 mM lithium for 24 h, or 500 μM AICAR for 1 h. Graphs depict band quantification (right). Total protein staining was used to normalize for protein loading. n = 3 independent experiments for each condition. Statistical significance was analyzed by one-way ANOVA with a Tukey *post hoc* test, ∗*p* < 0.05 and ∗∗*p* < 0.01. AICAR, 5-aminoimidazole-4-carboxamide; AMPK, 5′ AMP-activated protein kinase; MEF, mouse embryonic fibroblast; MIPS, myo-inositol-3-phosphate synthase; VPA, valproate; IP6K1, inositol hexakisphosphate kinase 1.

The inositol-depleting mood stabilizing drugs lithium and VPA have been shown to activate AMPK (24, 25, 27). To test the prediction that activation of AMPK by mood stabilizers induces nuclear localization of IP6K1, cells were treated with VPA (1 mM) or lithium (10 mM) for 24 h, and phosphorylated (active) AMPK was measured by Western blot (44). 5-aminoimidazole-4-carboxamide (AICAR), an AMP analog, was used as a positive control for AMPK activation. Treatment with either VPA or lithium increased phosphorylation of AMPK by ∼70% relative to the vehicle-treated control, while AICAR increased phosphorylation 3-fold (Fig. 6*A*). The total amount of AMPK protein was not altered by either treatment. The localization of IP6K1-GFP in cells treated with lithium or VPA for 24 h was examined by confocal microscopy. The nuclear localization of IP6K1 was increased by treatment with lithium or VPA (*p* < 0.05 and *p* = 0.07, respectively; Fig. 6*B*), and importantly, these treatments induced a 50% decrease in MIPS protein levels, similar to the effect of AICAR (Fig. 6*C*).

**Figure 7:**
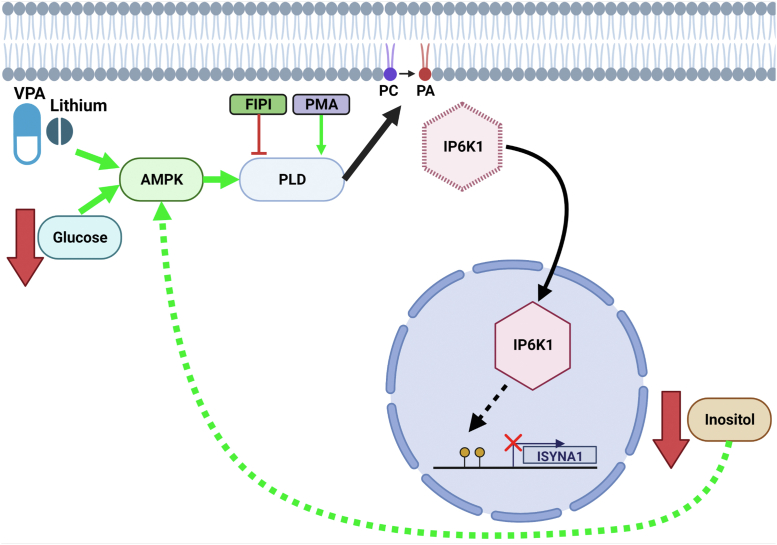
**Model of PA-mediated MIPS regulation and feedback.** PA can be synthesized through PLD, which converts PC into PA. PA synthesis in the plasma membrane leads to increased nuclear translocation of IP6K1 as a result of PA-IP6K1 binding. Once in the nucleus, IP6K1 induces methylation of the ISYNA1 gene promoter and inhibits MIPS expression (17). As a result, inositol levels are decreased. Inositol has been shown to negatively regulate AMPK activation (28); therefore, a decrease in inositol is predicted to lead to an increase in AMPK activity. AMPK can also be activated by low glucose or by treatment with the mood stabilizers VPA or lithium. Activated AMPK can in turn directly activate PLD (22). AMPK, 5′ AMP-activated protein kinase; IP6K1, inositol hexakisphosphate kinase; PA, phosphatidic acid; PC, phosphatidylcholine; PLD, phospholipase D; VPA, valproate; MIPS, myo-inositol-3-P synthase; IP6K1, inositol hexakisphosphate kinase 1. Figure created with BioRender.com.

Taken together, these findings supported a model in which PA regulates inositol synthesis by controlling localization of the negative regulator IP6K1 (Fig. 7). Increasing PA levels by activation of PLD (as a result of treatment with PMA or AMPK stimulation) results in translocation of IP6K1 to the nucleus and a decrease in MIPS protein levels.

## Discussion

Inositol is an essential nutrient for all eukaryotic life. It serves as a precursor for phosphoinositides, inositol phosphates, and inositol pyrophosphates, which mediate a myriad of vital cellular functions related to membrane integrity, metabolic regulation, and intracellular signaling and trafficking (1, 45, 46, 47). Considering its importance, it is surprising that very little is known about how inositol levels are regulated in higher eukaryotes. The current study provides the first in-depth description of a mechanism for inositol regulation in mammalian cells. Accordingly, binding of plasma membrane-derived PA to the inositol pyrophosphate biosynthetic enzyme IP6K1 mediates translocation of IP6K1 to the nucleus, where it represses MIPS expression and thereby decreases inositol synthesis. IP6K1 translocation is regulated by PA. Increasing PLD-derived PA through either pharmacological treatment or physiological conditions that activate AMPK results in increased nuclear localization of IP6K and MIPS repression (Fig. 7).

Interestingly, treating MEF cells with FIPI alone did not further suppress PA levels relative to the vehicle-treated control group (Fig. 1). However, a robust inhibitory effect of FIPI was observed in samples cotreated with PMA. The observed difference in PA levels between the ‘PMA alone’ and ‘PMA + FIPI’ groups confirmed that FIPI is indeed functional in our system and capable of suppressing PA synthesis. Therefore, the lack of a suppressive effect on PA for the ‘FIPI alone’ treatment suggests that 1) PLD activity (the target of FIPI) is maintained at a basal level in control cells and thus cannot be further inhibited and/or 2) compensatory mechanisms may upregulate alternative PA biosynthetic pathways (*e.g.*, *via* lysophosphatidic acid acyltransferase or diacylglycerol kinase) in ‘FIPI alone’ cells, resulting in no net change in overall PA levels. Suppression of PA synthesis in the ‘PMA + FIPI’ group suggests that PMA treatment (which acts on PKC upstream of PLD) potentiates the inhibitory effect of FIPI on PLD.

The exact nature of how PA influences IP6K1 localization is not clear, but the study by Yu *et al.* provided insight into this interaction (17). IP6K1 contains three functionally-relevant domains that influence PA-induced nuclear translocation and MIPS repression. These include a PA-binding domain, a conserved nuclear localization signal (NLS), and an ATP-binding domain required for catalytic activity of the protein (17). Deletion of either the PA-binding domain or NLS-containing domain of IP6K1 prevents nuclear translocation and repression of MIPS. Interestingly, deletion of the ATP-binding domain has no effect on nuclear translocation but does attenuate MIPS repression. This suggests that PA-mediated MIPS repression requires both PA-IP6K1 binding and IP6K1 catalytic activity.

Although the PA-binding domain of IP6K1 is indispensable for nuclear translocation, it is not clear whether this mechanism requires bound PA to accompany IP6K1 to the nucleus or whether translocation is an indirect effect of transient PA binding at the PM. Future studies will be aimed at elucidating these details. For example, it would be instructive to conduct liposome-binding assays to test the binding avidity between IP6K1 and physiologically representative membranes enriched with specific PA species. Conversely, transient PA binding at the PM could act to ‘prime’ IP6K1 for nuclear translocation, for example, by inducing a conformational change in the protein that exposes an otherwise obscured NLS or promotes interactions with additional transport proteins.

PA has been implicated in regulatory processes that control numerous aspects of cell growth, and specific PA species differ in their regulatory capacities (20). For example, mono- and di-unsaturated PA species are specifically involved in recruitment and docking of secretory vesicles, whereas poly-unsaturated PA uniquely participates in fusion pore expansion during exocytosis (48). The site of PA synthesis dictates the specific molecular species of PA produced (34, 35, 36). The finding that PA derived from the PM but not from the ER can induce nuclear translocation of IP6K1 (Fig. 3) suggests that binding is dependent on specific PA species synthesized predominantly in the PM *versus* the ER. Interestingly, the two major isoforms of PLD differ in their subcellular locations. PLD2 is localized primarily in the PM, whereas PLD1 is primarily found in organelle membranes, such as the nuclear membrane (38). Therefore, regulation of MIPS by PA may depend more on the activity of PLD2 than that of PLD1. However, we speculate that the molecular composition of newly synthesized PA is a more important/direct determinant for binding than the location of synthesis *per se*. Thus, other factors that influence the acyl chain composition of PA (*e.g.*, substrate availability and the biosynthetic enzyme/pathway utilized) may be relevant to the proposed model.

The role of IP6K1 catalytic activity in the repression of MIPS is not understood. IP6K1 catalyzes the conversion of IP_6_ to IP_7_, and IP_7_ has been shown to promote dissociation of the histone lysine demethylase JMJD2C from chromatin, resulting in increased trimethylation of histone H3 lysine 9 (H3K9me3) and transcriptional silencing of associated genes (16). Thus, IP6K1 activity may serve to silence MIPS by inducing methylation of the ISYNA1 promoter and/or increasing H3K9me3 (16).

It has been previously shown that AMPK is activated by IP_6_ and inhibited by IP_7_ (49). Thus, the ratio of nuclear to cytosolic IP6K1 may modulate levels of IP_6_ and IP_7_ and thereby regulate AMPK activation. Interestingly, treatment of cells with the inositol-depleting drugs lithium or VPA results in AMPK activation and IP6K1 nuclear translocation (Fig. 6). Lithium and VPA are known inhibitors of the inositol biosynthetic pathway; lithium uncompetitively inhibits inositol monophosphatase, and VPA indirectly inhibits MIPS by an unknown mechanism (11, 50, 51). The current findings suggest that, in addition to these previously described mechanisms, lithium and VPA may induce inositol depletion by activating AMPK and increasing PA production. Intriguingly, a recent study demonstrated that reduced inositol levels can directly activate AMPK in MEF cells (28). This suggests that there may be positive feedback between AMPK activation and inositol depletion wherein activated AMPK further reduces inositol levels by stimulating PLD-mediated synthesis of PA and in turn repressing MIPS expression *via* IP6K1 (Fig. 7). Furthermore, PA can also activate AMPK through the upstream kinase LKB1, suggesting that there may be additional feedback pathways between PA levels and AMPK activation (52). These scenarios are not mutually exclusive and may act in tandem to promote sustained activation of AMPK and inositol depletion.

Taken together, the current study delineates the first described mechanism for regulation of inositol synthesis in mammalian cells. This study demonstrates that transcription of the rate-limiting enzyme in the inositol biosynthetic pathway, MIPS, is negatively regulated by the abundance of a specific pool of PM-derived PA through the nuclear translocation of IP6K1 (Fig. 7). This study is, to our knowledge, the first to show regulation of MIPS protein levels in mammalian cells by treatment with the mood stabilizers VPA and lithium. This novel regulatory model has important implications for the treatment of bipolar disorder and other pathological conditions using inositol-depleting mood stabilizers, as the mechanism of action of these drugs may rely on feedback between inositol depletion and AMPK activation.

## Experimental procedures

### Cell culture, transfections, and treatments

WT and IP6K1-KO MEF cells (17) were cultured in Dulbecco’s modified Eagle’s medium (Gibco) supplemented with 10% fetal bovine serum (R&D Systems) and 1% penicillin-streptomycin (Gibco). For imaging, cells were grown either on glass coverslips or glass-bottomed 35 mm dishes (MatTek). Cells grown on coverslips were washed with PBS and fixed with a 4% solution of paraformaldehyde/sucrose in PBS. The coverslips were mounted onto microscope slides using ProLong Diamond Antifade Mountant with DAPI (Invitrogen). Cells grown in glass-bottom dishes were imaged without fixation. Constructs of GFP-PASS and RFP-PASS (32) were a kind gift from the laboratory of Dr Guangwei Du at the University of Texas Health Science Center at Houston. Plasmids for optogenetic induction of PLD: optoPLD-PM (Addgene plasmid # 140114), optoPLD-PM ‘dead’ (Addgene plasmid # 140061), optoPLD-ER (Addgene plasmid # 140060), and optoPLD-ER ‘dead’ (Addgene plasmid # 140059) (38) were constructed by the laboratory of Dr Jeremy Baskin from Cornell University, and purchased through Addgene. 500-1000 ng of plasmid were transfected using Lipofectamine 3000 transfection reagent (Thermo Fisher Scientific) following the manufacturer’s instructions. For cotransfections of two plasmids, a ratio of 1:1 was used. Transfection complexes were prepared in Opti-MEM (Thermo Fisher Scientific). All experiments were performed 24 to 48 h after transfection to ensure proper expression of the plasmids. PMA (Sigma-Aldrich, P8139), FIPI hydrochloride hydrate (Sigma-Aldrich, F5807), 18:1 Phosphatidic Acid (Avanti, 840875), lithium chloride (Sigma-Aldrich, L9650), valproic acid (Cayman, 13033), and N1-(β-D-Ribofuranosyl)-AICAR (Tocris, 2840) were used for cell treatments. Concentration and times of treatment are included in the corresponding figure legends.

### Optogenetic induction of PA synthesis

A blue light box was constructed with blue LED strips (1000bulbs.com - FLX-00036) surrounding an acrylic box with a lid. The LED strips were taped on the outside of the box with the lightbulbs facing in. The box was completely covered in black felt paper. The system was connected to a remote control. The box was placed inside a CO_2_ incubator. Plates of cells transfected with optoPLD plasmids were placed inside the light box and the incubator door was shut to allow for normal CO_2_ and humidity conditions. Cells inside the light box were stimulated with remotely controlled intermittent blue light in pulses of 5 s every 2 min, for a total of 20 to 30 min. After stimulation, cells were analyzed immediately by confocal microscopy.

### Protein extraction and Western blotting

Total cell lysates were obtained using a radioimmunoprecipitation assay buffer (Santa Cruz Biotechnology). Protein concentration was determined using a Bradford protein assay (Bio-Rad) against a bovine serum albumin standard. A total of 30 to 50 μg of total protein per sample was run on a 12% SDS-PAGE gel and transferred to a polyvinylidene difluoride membrane. Primary antibodies against MIPS (Invitrogen #PA5-44105, 1:500 dilution), p-AMPK (Cell Signaling #2531S, 1:1000 dilution), AMPK (Cell Signaling #2793S, 1:1000 dilution), and IP6K1 (GeneTex # GTX103949, 1:5000 dilution) were used. Corresponding HRP-tagged secondary antibodies (Invitrogen) or Alexa Fluor 488-labeled fluorescent secondary antibodies (Thermo Fisher) were used. The signal was detected using an iBright FL1500 imaging system (Thermo Fisher). iBright analysis software was used for band intensity quantification. No-stain protein labeling reagent (Invitrogen, A44717) was used to normalize for protein loading.

### Image analysis

All images were obtained on a Leica SP8 confocal microscope. Fluorescence quantification, analysis, and postacquisition detailing of images were performed with ImageJ.

### Statistical analysis

All graphs show average values ± SD. Statistical analyses were performed with GraphPad Prism software, version 6.1 (GraphPad). For column graphs, significant differences between experiments were assessed by an unpaired *t* test or a one-way ANOVA with a Tukey posttest, considering α as 0.05. For fluorescence graphs, significant differences between experiments were assessed by a Kolmogorov–Smirnov test, considering α as 0.05.

## Data availability

All data is contained within the article.

## Conflict of interest

The authors declare that they have no conflicts of interest with the contents of this article.
